# Real‐Life Indirect Case Matched Comparison of Dupilumab and Tezepelumab on Airway Oscillometry

**DOI:** 10.1111/all.70014

**Published:** 2025-08-19

**Authors:** Robert Greig, Rory Chan, Brian Lipworth

**Affiliations:** ^1^ Scottish Centre for Respiratory Research University of Dundee Dundee UK

**Keywords:** airway oscillometry, asthma, case matched, dupilumab, small airways dysfunction, tezepelumab

To the Editor,

Systemic biologics such as dupilumab and tezepelumab penetrating the peripheral airways may ameliorate small airway dysfunction (SAD) working on downstream and upstream type 2 inflammatory pathways, respectively. However, there are no indirect comparisons between these two drugs using airway oscillometry (AO) to assess peripheral airway resistance as heterogeneity of resistance between 5 Hz and 20 Hz (R5‐R20) and peripheral airway compliance as reactance area (AX) [1].

We report on retrospective outcomes of SAD in real‐life clinic patients with severe refractory asthma on either dupilumab or tezepelumab having been approved by NHS Tayside severe asthma multidisciplinary team. Patients were case matched in pairwise fashion on the basis of baseline R5‐R20 ≥ 0.10 kPa/L/s, as previously described by Chan et al. when indirectly comparing 3 months of treatment with dupilumab and benralizumab [2]. In addition, here, patients were also matched on biologic treatment duration, inhaled corticosteroid (ICS) dose, and BMI. All patients exhibited type 2 inflammation at baseline. Caldicott ethical approval was obtained prior to data collection, and patient consent was not required.

There were no significant differences between groups in baseline values as shown in Table S1.

Twenty‐two patients were case matched in pairs. Mean (95% CI) values for within‐treatment improvements for relative % change for dupilumab and tezepelumab were respectively R5‐R20: 53.92% (34.92, 72.92) *p* < 0.001 and 35.40% (12.25, 58.55) *p* < 0.01, AX: 60.53% (42.98, 78.07) *p* < 0.001 and 34.29% (12.93, 55.66) *p* < 0.01 (Figure 1). Mean (95% CI) differences between treatments comparing dupilumab vs. tezepelumab were 18.52% (3.97, 33.07) *p* < 0.05 for R5‐R20 and 26.23% (6.92, 45.55) *p* < 0.05 for AX. The within‐treatment responses alongside the mean between differences are shown in Table 1. Responder analysis of the change in absolute values showed eight patients on dupilumab exceeded the MCID proposed by Abdo et al. [3] for both R5‐R20 (≥ 0.06 kPa/L/s) and AX (≥ 0.65 kPa/L) compared to three patients on tezepelumab (*x* ^2^ = 4.55, *p* < 0.05).

**FIGURE 1:**
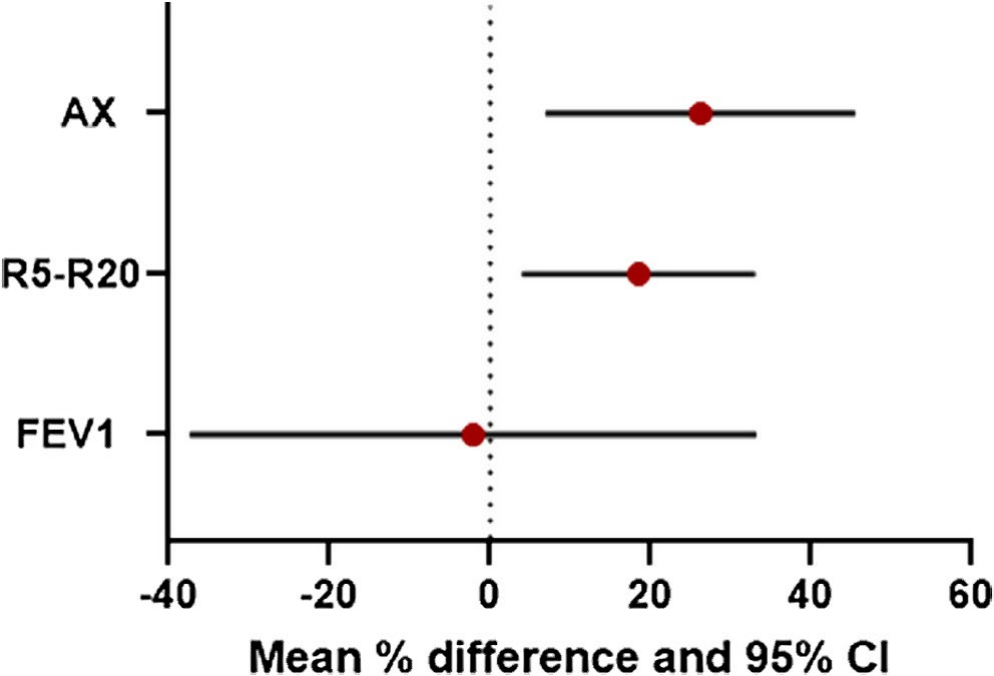
Mean and 95% CI for % difference between dupilumab and tezepelumab in large airways as FEV1 (NS) and in small airways as peripheral resistance (R5‐R20, *p* < 0.05) and peripheral compliance (AX, *p* < 0.05).

**TABLE 1:** Within treatment response to either dupilumab or tezepelumab as mean (95% CI) change from baseline, along with the mean between difference (95% CI).

Change in	Within dupilumab	Within tezepelumab	Difference between treatments
FEV1 (% predicted)	24.43 (4.11, 44.75)*	26.53 (−7.92, 60.97)	−2.09 (−37.41, 33.22)
FEF25–75 (% predicted)	66.13 (37.73, 94.54)***	47.17 (18.18, 76.16)**	18.96 (−13.55, 51.48)
R5–R20 (% change)	53.92 (34.92, 72.92)***	35.40 (12.25, 58.55)**	18.52 (3.97, 33.07)*
AX (% change)	60.53 (42.98, 78.07)***	34.29 (12.93, 55.66)**	26.23 (6.92, 45.55)*
Eosinophils (% change)	−40.64 (−75.06, −6.21)*	−77.38 (−87.59, −67.16)***	36.74 (3.18, 70.30)*
FeNO (% change)	−53.17 (−72.26, −34.07)***	−31.17 (−65.97, 3.63)	−22.00 (−60.50, 16.51)
ACQ	−1.50 (−2.38, −0.62)**	−1.18 (−2.03, −0.33)*	−0.32 (−1.79, 1.14)
AER	−2.36 (−4.39, −0.34)*	−2.45 (−4.19, −0.71)*	0.09 (−2.05, 2.23)

Abbreviations: ACQ: Asthma Control Questionnaire; AER: annualised exacerbation rate; AX: area under reactance curve; FEF25–75: forced expiratory flow rate between 25% and 75%; FeNO: Fractional exhaled nitric oxide; FEV1: forced expiratory volume in 1 s; R5–R20: resistance between 5 and 20 Hz.

* *p* < 0.05.

** *p* < 0.01.

*** *p* < 0.001.

The present study showed significantly greater improvements in response to dupilumab than tezepelumab for peripheral airway resistance and compliance reflecting smaller airways; despite there being no difference in FEV1 response reflecting larger airways. The lack of difference in FEV1 between dupilumab and tezepelumab has been previously reported in a more extensive indirect head‐to comparison [4].

We believe this is the first indirect case matched comparison between dupilumab and tezepelumab comparing the effects of upstream versus downstream cytokine blockade on oscillometry defined SAD. Both biologics attenuate signaling of IL‐4 and IL‐13, which are both expressed in human small airways smooth muscle [5], while IL‐13 is involved in mucus production [6]. Hence the mechanism for improving SAD with either drug could be due to either direct effects of small airways caliber or dissolving mucus plugs. The mean difference in FeNO between groups was 14 ppb, which was not significant; as such, we do not consider that higher baseline IL‐13 expression with dupilumab was likely to be clinically meaningful in determining small airways responses. Moreover, while the relative % suppression of FeNO was numerically greater with dupilumab, this was not statistically significant.

Chan et al. demonstrated superiority on R5‐R20 and AX when comparing 3 months of dupilumab to benralizumab, with the latter conferring no significant improvement; in turn, inferring that eosinophil depletion per se is not relevant to SAD^2^. Interestingly, our study shows that direct blockade of downstream IL‐4/13 to be more effective in improving oscillometry‐defined SAD compared to inhibiting IL‐4/13 via blocking upstream TSLP. One might perhaps postulate that despite TSLP blockade, there may be persistent upstream IL‐33 activity, in turn resulting in incomplete suppression of downstream type 2 cytokines.

Given the significant difference in treatment response between the two groups, one might perhaps expect that with a larger cohort this might become more pronounced along with tighter confidence intervals. Notably, both groups had near identical oscillometry baseline values and so had equal potential for improvement in their SAD. The next steps would be to undertake a direct head‐to‐head prospective study of these biologics to confirm our findings.

## Author Contributions

**Robert Greig:** data collection, statistical analysis and writing. **Rory Chan:** trial design and submission, review. **Brian Lipworth:** trial design, data interpretation and analysis, writing.

## Conflicts of Interest

Dr. Greig reports personal fees (talks) from AstraZeneca. Dr Chan reports personal fees (talks) and support attending ERS from AstraZeneca, personal fees (consulting) from Vitalograph, and personal fees (talks) from Thorasys.Dr Lipworth reports non‐financial support (equipment) from GSK; grants, personal fees (consulting, talks and advisory board), other support (attending ATS and ERS) and from AstraZeneca; personal fees (talks and consulting) from Sanofi, personal fees (consulting, talks and advisory board) from Circassia in relation to the submitted work; grants, personal fees (consulting, talks, advisory board), other support (attending ERS) from Teva, personal fees (talks and consulting), grants and other support (attending ERS and BTS) from Chiesi, personal fees (consulting) from Lupin, personal fees (consulting) from Glenmark, personal fees (consulting) from Dr Reddy, personal fees (consulting) from Sandoz; grants, personal fees (consulting, talks, advisory board), other support (attending BTS) from Boehringer Ingelheim, grants and personal fees (advisory board and talks) from Mylan outside of the submitted work; and the son of BJL is presently an employee of AstraZeneca.

## Supporting information


**Data S1:** all70014‐sup‐0001‐DataS1.docx.

## Data Availability

The data that support the findings of this study are available from the corresponding author upon reasonable request.
