# Supplementary material for: Real‐Life Indirect Case Matched Comparison of Dupilumab and Tezepelumab on Airway Oscillometry
Source: Allergy. 2025 Aug 19;81(1):260–2. doi: 10.1111/all.70014 (PMC12773657; doi:10.1111/all.70014)
Supplement: Supplementary file 1 — Data S1: all70014‐sup‐0001‐DataS1.docx. [file ALL-81-260-s001.docx]

**Supplement**: **Real-life indirect case matched comparison of dupilumab and tezepelumab on airway oscillometry**

**Supplementary methods**

Patients were identified from the NHS severe asthma multidisciplinary team meeting who had been commenced on biologics between April 2021 (dupilumab) / January 2024 (tezepelumab) and October 2024. This involved initially screening 47 patients on dupilumab and 66 patients on tezepelumab. From this cohort, 28 patients on dupilumab and 20 patients on tezepelumab were identified with oscillometry defined small airways dysfunction (SAD). This was calculated by determining the heterogenicity in resistance between the whole airway at 5Hz (R5) and the large airway resistance measured at 20Hz (R20). SAD here was defined as an R5-R20 ≥0.10kPa/L/s^1^. Patients were then matched in pairs according to oscillometry defined SAD values. Patients were also matched on BMI^2^, ICS dose and treatment duration**.** 22 patients ended up meeting the pairwise matching criteria which were then for the purpose of comparison between biologics.

Oscillometry was measured in triplicate using IOS Masterscreen (Carefusion Hoechberg, Germany) according to the ERS technical standards^3^. Fractional exhaled nitric oxide (FeNO) was obtained using NIOX Vero (NIOX, Oxford, UK) following the manufacturer's instructions and ERS guidelines^4^. Spirometry (Micromedical, Chatham, UK) was performed in triplicate according to the ERS guidelines. The 6-point asthma control questionnaire (ACQ) was used to measure symptom control. SPSS version 29 was used for statistical analysis. Paired Students T-tests were applied with a two-tailed alpha error of 5% to identify mean differences.

The baseline demographics are shown in table E1. There were no active smokers included in our cohort. There were 3 and 4 ex-smokers within the dupilumab and tezepelumab groups respectively. All patients were taking combined inhaled corticosteroid and long-acting beta-2 agonists with 6 patients on dupilumab and 7 patients on tezepelumab also taking long acting muscarinics throughout. 9 patients on dupilumab and 4 on tezepelumab were taking extra fine particle inhalers which they were established on prior to commencing a biologic and remained throughout the study period. Prior to consideration of a biologic, all patients were assessed to ensure >80% adherence to their inhaled therapies. Nasal polyps were present in 8 patients on dupilumab and 3 on tezepelumab; 1 patient on tezepelumab had atopic dermatitis and no patients on dupilumab. 2 patients on dupilumab had a psychiatric history compared to 1 tezepelumab patient.

**Table E1:** Baseline demographics for severe asthma patients matched on small airways defunction characterised by R5-R20 taking either dupilumab or tezepelumab. There were no significant differences between baseline values.

|  | Dupilumab | Tezepelumab |
| --- | --- | --- |
| Age (years) | 59.09 (48.79, 69.39) | 56.09 (43.83, 68.36) |
| BMI (kg/m^2^) | 31.24 (28.52, 33.96) | 30.08 (27.85, 32.31) |
| Female (%) | 45% | 45% |
| BDP equivalent ICS dose (μg) | 1600 (1302, 1898) | 1527 (1334, 1720) |
| FEV1 % predicted | 70.56 (56.57, 84.56) | 76.58 (64.36, 88.80) |
| FEF25-75 % predicted | 42.58 (28.98, 56.18) | 48.83 (29.85, 67.80) |
| FVC % predicted | 86.47 (71.24, 101.70) | 83.13 (61.95, 104.31) |
| R5-R20 (kPa/L/s) | 0.209 (0.148, 0.270) | 0.209 (0.148, 0.270) |
| AX (kPa/L) | 2.18 (1.30, 3.06) | 2.24 (1.28, 3.21) |
| Eosinophils (cells/uL) | 739 (491, 988) | 654 (460, 847) |
| FeNO (ppb) | 61.64 (43.53, 79.75) | 47.64 (27.31, 67.97) |
| ACQ | 2.53 (2.01, 3.04) | 3.16 (2.18, 4.14) |
| Annualised exacerbation rate | 2.81 (0.70, 4.94) | 4.00 (1.71, 6.29) |
| Duration on biologic (months) | 7.86 (6.15, 9.58) | 8.27 (7.31, 9.23) |

**Supplementary References**

1. Chan R, Lipworth BJ. Determinants of asthma control and exacerbations in moderate to severe asthma. *J Allergy Clin Immunol Pract* 2022; **10**(10): 2758-60 e1.

2. Chan R, Lipworth B. Clinical impact of obesity on oscillometry lung mechanics in adults with asthma. *Ann Allergy Asthma Immunol* 2023; **131**(3): 338-42 e3.

3. King GG, Bates J, Berger KI, et al. Technical standards for respiratory oscillometry. *Eur Respir J* 2020; **55**(2).

4. Horvath I, Barnes PJ, Loukides S, et al. A European Respiratory Society technical standard: exhaled biomarkers in lung disease. *Eur Respir J* 2017; **49**(4).
